# Griscelli Syndrome in Two Siblings with Silvery Hair: A Case Report

**DOI:** 10.31729/jnma.v63i292.9262

**Published:** 2025-12-31

**Authors:** Hanniyah Khwaja, A.R. Rajan, Nitin Lingayat, Shweta Dhasal

**Affiliations:** 1 Symbiosis Medical College for Women & Symbiosis University Hospital & Research Centre, Symbiosis International (Deemed University), Pune, India; 2 Department of Paediatrics, Symbiosis Medical College for Women & Symbiosis University Hospital & Research Centre, Symbiosis International (Deemed University), Pune, India

**Keywords:** *albinism*, *clumpedmelanosomes*, *griscelli syndrome*, *lymphohiSiocytosis*

## Abstract

Griscelli syndrome (GS) is an uncommon disorder characterized by partial albinism, which gives hair a silvery-grey sheen and variable immunodeficiency or neurological impairment, with pancytopenia, immune dysfunction, hepatosplenomegaly, neurological impairment, hypogammaglobulinemia, and variable cellular immunodeficiency. Three variants GS1, GS2 and GS3 have been described in different phenotypes of the disease with varying presentation.

We present two neonates, born two years apart, to parents with third-degree consanguinity. Both had features of partial albinism and neutropenia at birth. Microscopy of hair showed characteristic large aggregates of pigment granules dispersed irregularly along the hair shaft. Given their family history and high clinical suspicion, a diagnosis of Griscelli syndrome was made. Early diagnosis of Griscelli syndrome can offer treatment options like Bone Marrow Transplant and prevent fatal complications like hemophagocytic lymphohistiocytosis. Supportive management during transplantation comprises of antimicrobial therapy, immunoglobulin replacement, and vigilant monitoring for complications like graft versus host disease.

## INTRODUCTION

Griscelli syndrome, characterised by pigmentary dilution of skin and hair, neurological deficits, infections, and immunodeficiency is a rare autosomal recessive disorder of which only 150 cases have been reported as per the Genetic and Rare Diseases Information Centre.^1,2,3,4^ It results from mutations in MYO5A (GS1), RAB27A (GS2), or MLPH (GS3) genes on chromosome 15q21, affecting melanosome transport in melanocytes.^4,5,6,7^ While GS3 presents only with pigmentary issues, GS1 includes neurological symptoms, and GS2 features severe immunodeficiency and hemophagocytic lymphohistiocytosis.^8^ GS2 is often fatal within the first decade of life. This case report discusses two siblings diagnosed with Griscelli syndrome.

## CASE REPORT

### CASE 1

A female neonate was delivered vaginally at term, weighing 2800 grams, after an uneventful antenatal period, to parents with third-degree consanguinity. There was no intrapartum digress and the baby cried spontaneously after birth. Head to foot examination at birth, revealed features of hypopigmentation and silvery hair to be present over the scalp and body. The baby had no immediate neonatal complications. There were no obvious congenital deformities. The vital parameters were normal with heart rate 130 per minute (100-160/min) and respiratory rate of 48 per minute (<60/min). The family history and the phenotypical features prompted us to investigate further.

Physical Examination: General examination revealed silvery grey patches of scalp hair, eyebrows, eyelashes, and vellus hair all over the body. Examination of the eye revealed a normal brown-black iris. There was no hepatosplenomegaly. The neurological examination, including neonatal reflexes was normal. The baby had physiological jaundice which resolved spontaneously. Investigations are shown in (Table 1) below.

**Table 1:** Haematological investigation of patient.

Haematological parameter	Observed Value	Normal Range
Haemoglobin	18 gm%	13.4-19.9 gm%
Total Leucocyte	6500/mm3 count	9000-30000/mm3
Polymorphs	12%	55-70%
Reticulocyte count	1%	3-5 %

The peripheral blood smear revealed marked neutropenia (12%). There were no giant cytoplasmic granules in leukocytes. The hepatic, renal parameters and serum electrolytes were normal. Septic screen, including blood-culture, urine examination, coagulation profile and immunoglobulin profile was normal. Abdomen and cranial sonography were normal.

**Figure 1:**
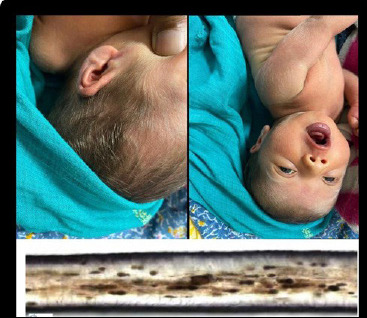
Sparsely distributed silvery grey hair over the scalp and body. On microscopy, large pigment clumps melanocytes with sparsely pigmented adjacent keratinocytes.

The hair shaft microscopy revealed large clumps of pigment granules distributed irregularly along the width of the hair shaft (Figure 1).

**Figure 2:**
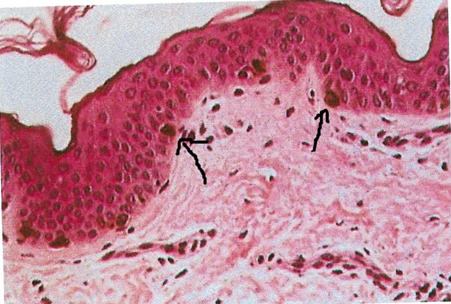
Skin biopsy reveals aggregates of melanin in the basal layer of the epidermis(black arrow)^11^.

Skin biopsy done from a hypopigmented area over the back on microscopy revealed large coarse aggregates of melanin in the basal layer of the epidermis, showing reactivity with the Fontana-Masson stam (Figure 2).

A clinical suspicion of Griscelli syndrome was entertained. Neurosonography and echocardiogram were done which were normal.

Genetic studies: A whole genome sequencing revealed a homozygous missense variant located in exon 3 of the RAB27A gene. These findings are indicative of Griscelli Syndrome type 2. Follow up in the neonatal period was unremarkable with normal weight gain. Our case was diagnosed in the neonatal period and thus had not developed the severe accelerated phase of hemophagocytic lymphohistiocytosis and its severe complications.

### CASE 2

A female neonate was born to the same (third-degree consanguineous) couple 2 years ago, after an uneventful antenatal period. The baby had hypopigmentation and silvery hair on the scalp and body. The neonatal period was unremarkable with no history of NICU stay. There was a history of recurrent (2-3) episodes of fever and respiratory symptoms (increased chest activity and poor feeding) after 2 months of age for which antibiotic (amoxycillin) was exhibited. At 4 months of life, there was a febrile episode followed by abdominal distension, respiratory distress, poor feeding, lethargy and features suggestive of sepsis. She was admitted and diagnosed as bacterial pneumonia with septic shock and managed with ventilatory support, antibiotics (Injection Ceftriaxone @100mg/kg/day), inotropes and supportive care. The baby however had a fatal outcome.

At birth both babies only had silvery hair and hypopigmented skin. The history of adverse outcome in first child after recurrent infections points to a possible immunodeficient state. However, she was not evaluated. The present baby was diagnosed with Griscelli syndrome Type 2 in genomic sequencing and was hence referred for Bone marrow transplant to improve survival.

Genetic counselling was given to the parents explaining a 25% chance of recurrence and were advised exome sequencing to detect carrier state but due to financial constraints the same was not carried out.

## DISCUSSION

Less than 150 cases of Griscelli syndrome have been reported in literature thus far. Earlier case reports are of single cases .^1,5,8,9^ Our case was detected in the neonatal period giving hope for timely treatment in the form of hematopoietic stem cell transplantation. The phenotypic appearance (hypopigmentation and light silvery grey hair on scalp and body) and hair microscopy revealing typical large clumps of pigments in the hair shaft of the patient, made us entertain the diagnosis of Griscelli Syndrome (GS). The differential diagnosis considered in view of the hypopigmentation and silvery hair were Chediak-Higashi syndrome and Elejalde syndrome.

In Chediak-Higashi syndrome, uniform pigment distribution in normal hair shafts with punctuating pattern is seen along with presence of neutrophilic giant lysosomal inclusion bodies. Elejalde disease, presents with silvery hair and neurological deficit and was excluded by hair shaft microscopy.

Whole genome sequencing revealed a homozygous missense variant located in exon 3 of the RAB27A gene, resulting in a threonine-to-isoleucine substitution at codon 23, confirming the diagnosis of Griscelli Syndrome Type 2.

Early hematopoietic stem cell transplantation (HSCT) remains the most effective therapeutic option in view of the high risk of morbidity and mortality following hemophagocytic lymphohistiocytosis (HLH) in Griscelli syndrome. Reports describe successful long term survival outcomes with allogeneic bone marrow transplantation from an HLA-matched sibling donor. In our case absence of a sibling delayed early HSCT. The child is immunized for age and on regular follow up pending availability of HLA matched donor.

Griscelli Syndrome is a rare autosomal recessive immunodeficiency disorder, characterized by pigmentary dilution of skin and hair, recurrent multisystemic infections, neurologic disability, hypogammaglobulinemia and variable cellular immunodeficiency.^1^

Three subtypes (GS1, GS2, and GS3) have been distinguished based on mutations in genetic loci (myosin VA, Ras-related protein Rab-27A, and melanophilin, respectively). GS1 presents with neurologic impairment without immunological dysfunction while GS2 presents with immunological dysfunction, hepatosplenomegaly and multisystem involvement and GS3 presents with only hypomelanosis. Whole genome sequencing, which identifies a homozygous missense mutation in exon 3 of the RAB27A gene, narrowed the diagnosis to Griscelli Syndrome type 2. This pathogenic variant results in the substitution of threonine with isoleucine at codon 23 and is consistent with the molecular profile of GS type 2.

Palliative treatments are suggested in GS1 patients while GS3 patients require no treatment, and it has an exceptionally great prognosis.^9^ However, GS-II can cause hemophagocytic lymphohistiocytosis (HLH), leading to activated T lymphocytes and macrophages; these can damage body organs and tissues, causing life-threatening complications. T-cell dysfunction, hypogammaglobulinemia and neutropenia are known to be related to Griscelli Syndrome.^1^ Most patients have recurrent and severe fungal, viral, and bacterial infections. Hematopoietic stem cell transplantation is the only treatment for these patients.^10^ Supportive management during transplantation comprises of antimicrobial therapy, immunoglobulin replacement, and vigilant monitoring for complications like graft versus host disease. This highlights the importance of early recognition of Griscelli’s syndrome and prompt referral for transplantation.

Genetic counselling and screening for carrier state in the parents by exome sequencing can improve predictive counselling.
